# Study of the Relationship between Taste Sensor Response and the Amount of Epigallocatechin Gallate Adsorbed Onto a Lipid-Polymer Membrane

**DOI:** 10.3390/s150306241

**Published:** 2015-03-13

**Authors:** Yuhei Harada, Yusuke Tahara, Kiyoshi Toko

**Affiliations:** 1 Graduate School of Information Science and Electrical Engineering, Kyushu University, Nishi-ku, Fukuoka 819-0395, Japan; E-Mails: y.harada@nbelab.ed.kyushu-u.ac.jp (Y.H.); toko@ed.kyushu-u.ac.jp (K.T.); 2 Research and Development Center for Taste and Odor Sensing, Kyushu University, Nishi-ku, Fukuoka 819-0395, Japan

**Keywords:** taste sensor, lipid-polymer membrane, CPA value, astringent substance, epigallocatechin gallate

## Abstract

A taste sensor using lipid-polymer membranes has been developed to evaluate the taste of foods, beverages and medicines. The response of the taste sensor, measured as a change in the membrane potential caused by adsorption (CPA), corresponds to the aftertaste felt by humans. The relationships between the CPA value and the amount of adsorbed taste substances, quinine and iso-α acid (bitterness), and tannic acid (astringency), have been studied so far. However, that of epigallocatechin gallate (EGCg) has not been clarified, although EGCg is abundantly present in green tea as one of its astringent substances. This study aimed at clarifying the response of the taste sensor to EGCg and its relationship with the amount of EGCg adsorbed onto lipid-polymer membranes. The lipid concentration dependence of the CPA value was similar to that of the amount of adsorbed EGCg, indicating a high correlation between the CPA value and the amount of adsorbed EGCg. The CPA value increased with increasing amount of adsorbed EGCg; however, the CPA value showed a tendency of leveling off when the amount of adsorbed EGCg further increased.

## 1. Introduction

The five basic taste qualities which humans perceive with the tongue include sourness, saltiness, bitterness, sweetness, and umami. These tastes are perceived by the taste buds consisting of taste cells [1]. Astringency is perceived as both the sense of taste and pain, and is recognized as a kind of bitterness in the meaning of sense of taste. Astringency affects individual preference in foods and beverages, and hence evaluation of astringency is important for the development of new products and marketing in the food industry [2]. The main method of evaluating taste are sensory tests in which sensory panelists actually taste samples to evaluate them; however, these tests have some problems such as low objectivity and reproducibility as well as the stress possibly imposed on panelists. Against this background, sensors that can objectively evaluate taste including astringency have attracted considerable attention [3,4,5,6,7].

A taste sensor comprising potentiometry-based sensor electrodes using lipid-polymer membranes, each of which is selectively responsive to each taste, has been developed to evaluate the taste of foods and beverages [3,4,5,8]. A lipid-polymer membrane made of lipids, plasticizers and a polymer is used as the taste sensing part. The output of the taste sensor is a change in the membrane potential generated by the interactions between the lipid-polymer membrane and the taste substances. These interactions are mainly the electrostatic interaction and the hydrophobic interaction. The kind and the quantity of lipids and plasticizers are changed and controlled so as to respond selectively to each taste quality.

The properties common to the taste sensor and electronic tongues (e-tongues) are both using semi-selective sensor electrodes and measuring liquid samples. Concepts of the taste sensor are as follows: (I) the taste sensor must respond consistently to the same taste like the human tongue; (II) the taste sensor threshold must be the same as the human taste threshold; (III) there must be a clearly defined unit of information from the taste sensor; (IV) the taste sensor must detect interactions between taste substances. The taste sensor can discriminate and quantify samples into the five basic taste qualities [3,4,5]. On the other hand, e-tongues aim to discriminate and analyzer liquid samples such as foods and beverages using several kinds of electrodes which have different responding characteristics together with statistical analysis such as principal component analysis (PCA) and neural network techniques [5,6,9,10].

**Figure 1:**
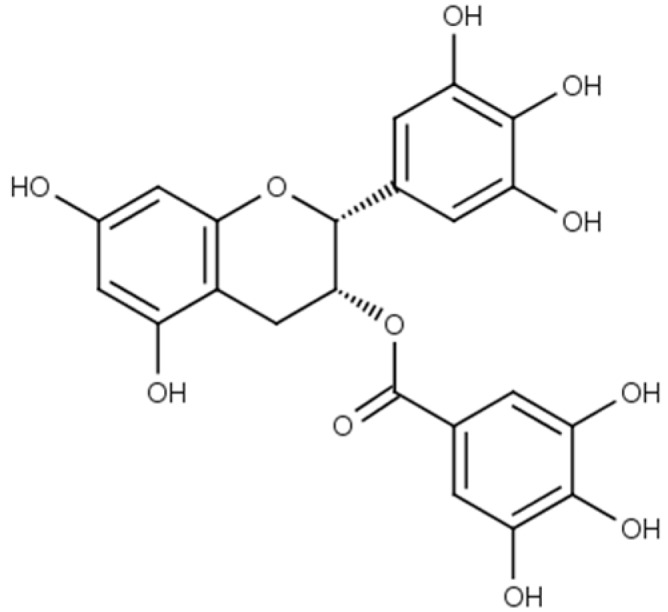
Structure of epigallocatechin gallate.

The membrane potential is changed with astringent substances adsorbed onto the lipid-polymer membrane by the hydrophobic interaction [11]. In the taste sensor, the change in membrane potential caused by adsorption (abbreviated by CPA) is used as an index of the aftertaste [12]. We have researched the relationship between the CPA value and the amount of the adsorbed taste substances quinine [13] and iso-α acid [14] to show bitterness, and tannic acid [11] to show astringency. Study of the relationship between these two quantities (*i.e.*, CPA value and adsorption amount) can clarify the mechanism of sensor response to adsorptive taste substances, and will also be useful for improving the taste sensor. In this study, therefore, we measured the CPA value for epigallocatechin gallate (EGCg, Figure 1), an astringent substance, using the taste sensor and examined its relationship with the amount of EGCg adsorbed onto the lipid-polymer membrane. EGCg is the polyphenol known as catechin found in green tea, and it has attracted attention for its potential health benefits. Although several other catechins are also found in lower quantity in green tea, EGCg accounts for more than 50% and hence this fact suggests that EGCg is responsible for the majority of the potential health benefits [15,16].

## 2. Experimental Section

### 2.1. Reagents

Tetradodecylammonium bromide (TDAB) and dioctylphenyl phosphonate (DOPP) were purchased from Sigma-Aldrich, Inc. (St. Louis, MO, USA). Polyvinyl chloride (PVC) and epigallocatechin gallate (EGCg) were purchased from Wako Pure Chemical Industries, Ltd. (Osaka, Japan). Potassium chloride (KCl) and tartaric acid were purchased from Kanto Chemical Co., Inc. (Tokyo, Japan). All aqueous solutions were prepared with distilled water.

### 2.2. Lipid/Polymer Membranes

**Figure 2:**
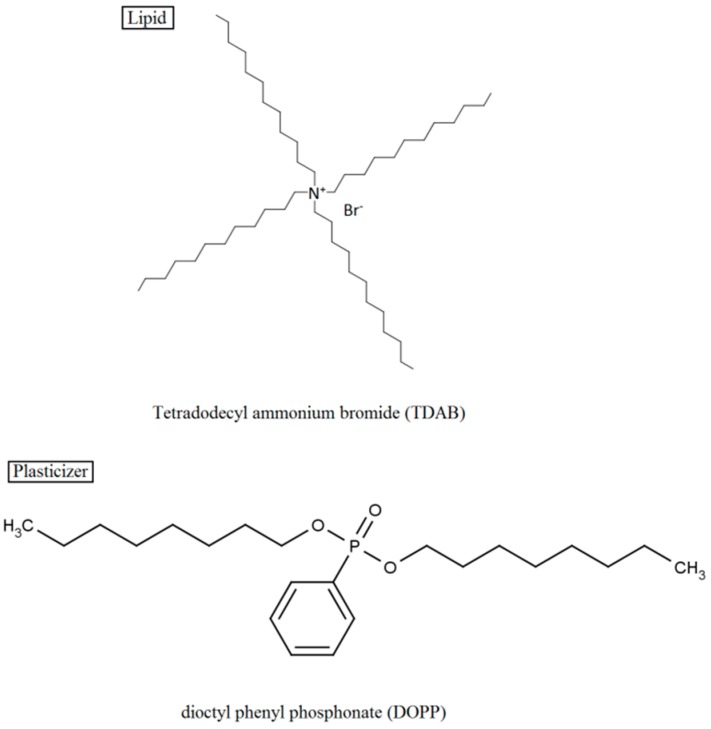
Structure of the lipid and plasticizer used for the lipid-polymer membranes.

We used lipid-polymer membranes composed of 0.01–20 wt% TDAB as the lipid, 0.6 mL DOPP as the plasticizer (Figure 2) and 800 mg PVC as the polymer. The TDAB concentration is shown as wt% in comparison with the PVC content. We selected these lipid-polymer membrane components because this lipid-polymer membrane has already been used in a commercialized taste sensor.

Previously, we reported that the CPA values of the sensor showed high selectivity. The response to salty (270 mM KCl), sour (0.27 mM tartaric acid) and umami (10 mM monosodium glutamate, 10 mM disodium 5'-inosine monophosphate and 10 mM disodium 5'-guanosine monophosphate), bitter (0.1 mM quinine, 0.1 mM cetirizine, 0.1 mM hydroxyzine and 0.1 mM bromhexine) and sweet substances (1 M sucrose) were smaller than −3 mV [4]. The procedures for fabricating the membranes follow those used in the previous studies [3,4,5,8]. These membranes are positively charged because TDAB is positively charged in aqueous solution. They were designed to contain different lipid concentrations, because the properties of the membranes, *i.e.*, the surface charge density and hydrophobicity, which affect the CPA value and the amount of adsorbed astringent substances, depend on the lipid concentration.

### 2.3. Measurement of CPA Value Using Taste Sensor

**Figure 3:**
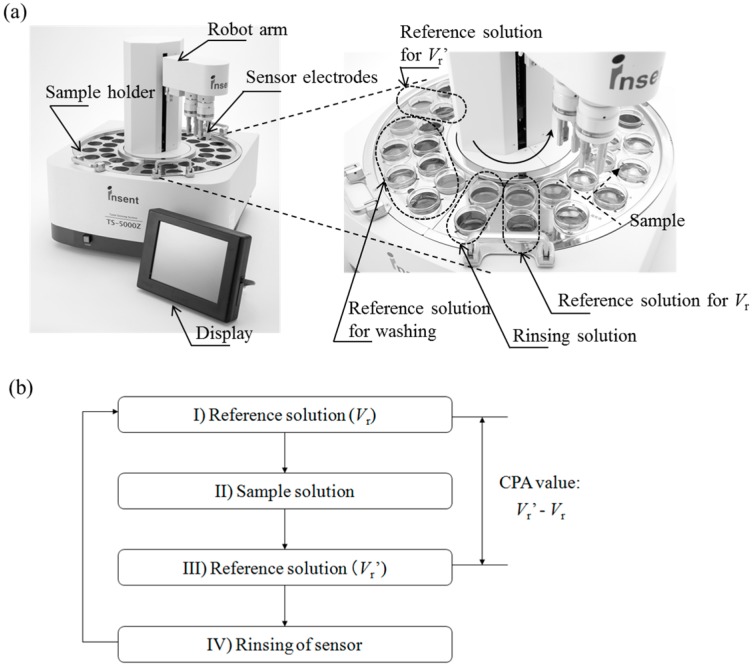
Schematic of the taste sensing system and CPA value measurement procedure. The TS-5000Z taste sensing system (470 mm × 530 mm × 510 mm, 26 kg) and sample holder (**a**); measurement procedure for the CPA value (**b**).

The TS-5000Z taste sensing system (Intelligent Sensor Technology, Inc., Kanagawa, Japan) was used for the CPA measurement. The taste sensor has several sensor electrodes, to each of which a different lipid-polymer membrane is attached, and a reference electrode (Ag/AgCl electrode). It measures the changes in the membrane potential that are generated when the sensor electrodes are immersed in a sample solution. The sample solution is EGCg (0.01–2 mM), and the solvent is a reference solution comprising 30 mM KCl and 0.3 mM tartaric acid. The measurement procedure [4,5,11,13,14] is as follows (Figure 3). At first, the sensor electrode is immersed in the reference solution, and the membrane potential for the reference solution, *V*_r_, is measured. Next, the sensor electrode is immersed in the sample solution (EGCg solution). After that, the sensor electrode is again immersed in the reference solution, and the membrane potential for the reference solution, *V*_r_', is measured. Then the difference between *V*_r_' and *V*_r_, *i.e.*, *V*_r_'–*V*_r_, is defined as the CPA value. At last, the membrane is rinsed with a rinsing solution consisting of 100 mM KCl, 10 mM KOH, 30 vol% ethanol. This procedure is repeated five times for each sample, and the average of the CPA values except the first measurement value is used as the CPA value of each sample.

### 2.4. Measurement of Amount of Adsorbed EGCg

**Figure 4:**
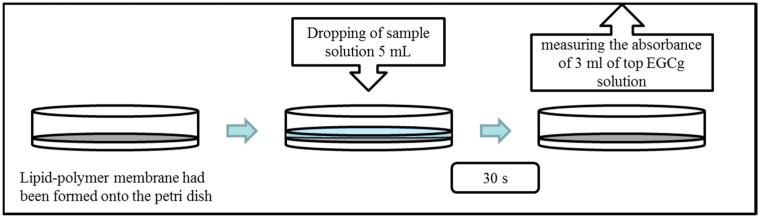
Measurement procedure of the amount of adsorbed EGCg.

The amount of EGCg adsorbed onto the lipid/polymer membrane was measured using an UV-visible spectrometer (UV-1800, Shimadzu Corp., Kyoto, Japan). The absorbance of EGCg solution, the concentration of which is known, was measured to obtain a calibration curve. The measurement procedure [11,13,14] is shown in Figure 4. The lipid-polymer membrane was formed in a Petri dish, and then five mL of EGCg solution of known concentration was added onto the Petri dish. After 30 s, 3 mL of the top EGCg solution was taken from the Petri dish and the absorbance of this solution was measured after diluting with reference solution to appropriate absorbance values for the spectrometer (two, five or ten times). The EGCg concentration in the measured solution was calculated by the calibration curve. The amount of adsorbed EGCg was calculated from the difference of the concentration of the EGCg before and after added. This value divided by the size of the Petri dish was the amount of EGCg adsorbed per square centimeter.

## 3. Results and Discussion

### 3.1. Measurement of CPA Value Using Taste Sensor

**Figure 5:**
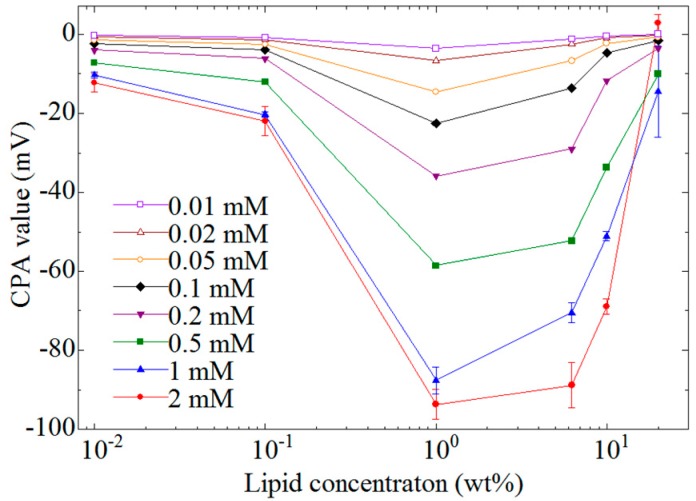
Relationship between CPA value and lipid concentrations. The concentrations of EGCg are 0.01–2 mM. Data are expressed as mean ± SD (*n* = 16).

The CPA values for EGCg using six kinds of membrane containing different lipid concentrations were measured. Figure 5 shows the lipid concentration dependence of the CPA value. The magnitude the of CPA value increases with increasing lipid concentration in the membrane, and showed the highest score around 1 wt%; however, the CPA value decreased with the lipid concentration over 1 wt%.

### 3.2. Measurement of Amount of Adsorbed EGCg

**Figure 6:**
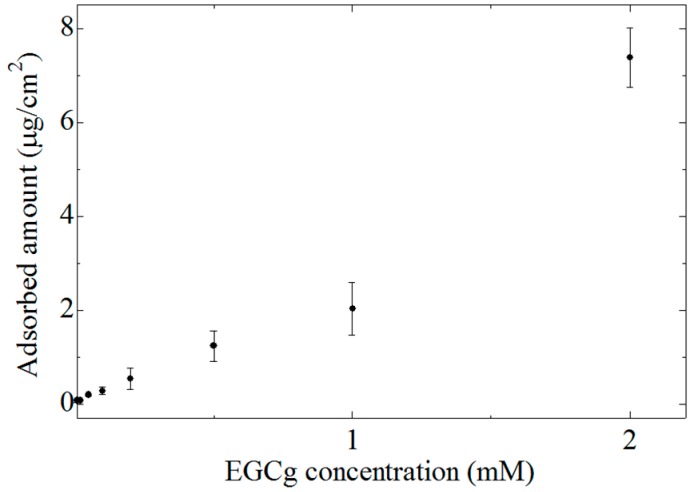
EGCg concentration dependence of the amount of adsorbed EGCg. Data are expressed as mean ± SD (*n* = 9).

Figure 6 shows the EGCg concentration dependence of the amount of EGCg adsorbed onto the lipid-polymer membrane. A lipid/polymer membrane with 1 wt% lipid concentration, where the highest CPA value as shown in Figure 5, was used in this measurement. The amount of adsorbed EGCg was 0.07–7.4 µg/cm^2^ and increased with increasing EGCg concentration.

**Figure 7:**
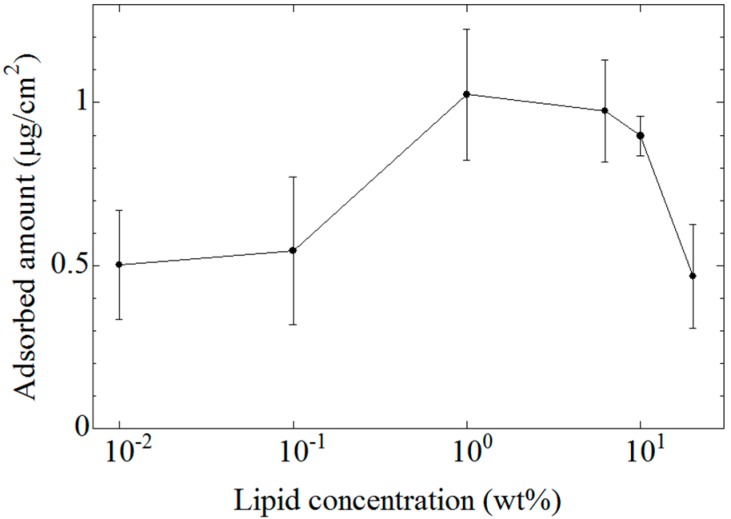
Lipid concentration dependence of the amount of adsorbed EGCg. Data are expressed as mean ± SD (*n* = 9). The lipid concentration is shown by wt % in comparison with the PVC content.

Figure 7 shows the dependence of the amount of adsorbed EGCg on the lipid concentration of the membrane. We used 0.2 mM EGCg solution in this measurement. The amount of adsorbed EGCg increased with increasing lipid concentration of the membrane, and showed the highest score around 1 wt%; however, the amount of adsorbed EGCg decreased with further increases of the lipid concentration. The lipid concentration dependence of the amount of adsorbed EGCg was similar to that of the CPA value.

The CPA value is generated when taste substances are adsorbed onto the surface of a lipid-polymer membrane and then change the charge density of the membrane surface [4,5,11,13,14]. As the lipid concentration of the lipid-polymer membrane increases, the surface charge density of the membrane increases because lipid molecules are positively charged, and hence the hydrophobicity of the membrane decreases. When the surface charge density of the membrane increases, the electrostatic interaction which attracts EGCg in the solution to the membrane surface becomes stronger. Then, the amount of EGCg adsorbed onto the membrane increases, which results in an increasing CPA value.

The distribution coefficient (log*D*) of EGCg is around 3.0 at acidic and neutral pH values in the present experiment. Therefore, EGCg can be adsorbed into the hydrophobic part of the membrane. The amount of EGCg adsorbed onto the membrane surface or into the membrane interior decreases when the hydrophobicity of the membrane decreases. As a result, the CPA value decreases together with decreasing hydrophobicity. Therefore, a peak should exist in the lipid concentration dependence of the CPA value.

### 3.3. CPA Value and Amount of Adsorbed EGCg

**Figure 8:**
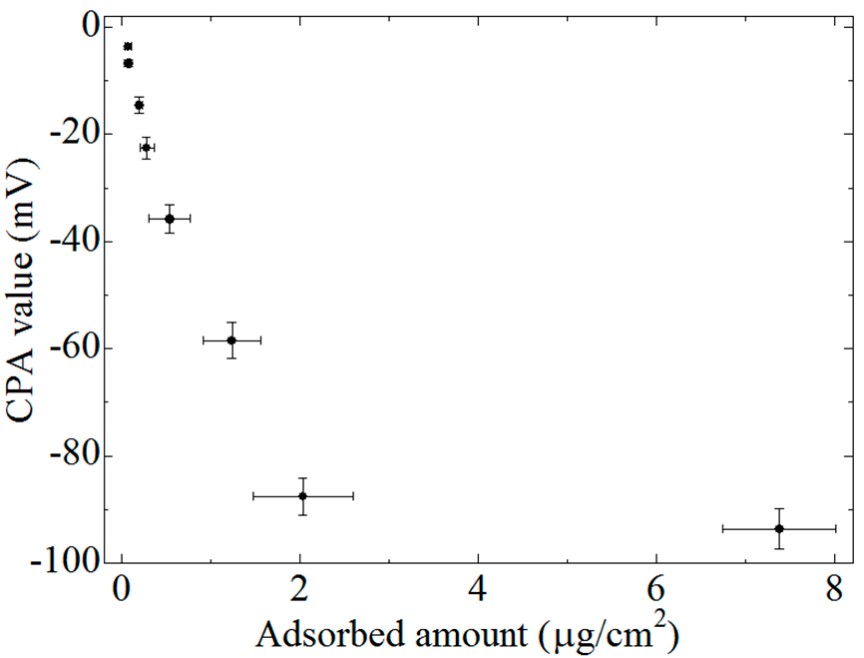
Relationship between the CPA value and the amount of adsorbed EGCg. Data are expressed as mean ± SD (CPA value, *n* = 16; adsorbed amount, *n* = 9). The CPA values ware used same data of Figure 3 (1 wt % lipid concentration).

Figure 8 shows the relationship between the CPA value and the amount of EGCg adsorbed onto the lipid/polymer membrane with 1 wt % lipid concentration. The CPA values were the same data of Figure 3 (1 wt% lipid concentration). The error bars increased with increasing amount of absorbed EGCg, whichwas caused by the dilution procedure. The CPA value increased with increasing amount of adsorbed EGCg. However, the CPA value showed a tendency to level off when the amount of adsorbed EGCg exceeded 2 μg/cm^2^. This phenomenon can be explained as follows: when the amount of adsorption is roughly 2 μg/cm^2^, the density of EGCg molecules adsorbed onto the membrane is estimated to be 2.63 × 10^15^ /cm^2^. When the predicted value of maximal projection area of EGCg is calculated using MarvinSketch (ChemAxon Ltd., Budapest, Hungary), it amounts to roughly 100 Å^2^. Hence, the maximum number of EGCg molecules which can be adsorbed per square centimeter can be estimated as 10^14^. Therefore, we can consider that as a rough estimate at least about 26 layers of EGCg molecules are deposited on the surface and interior of the membrane. The multilayered adsorption prevents EGCg from being negatively charged, and causes neutralization; the suppression of dissociation and the electrostatic screening by paired ions occur. In addition, EGCg molecules adsorbed into the membrane interior have little effect on the membrane surface electric charge, and hence they do not contribute to the change in the membrane potential. Thus, the rate of increase of the CPA value decreased.

In previous studies [11,13,14], the amount of adsorption showed a maximum at a certain lipid concentration due to increasing electrostatic interactions and decreasing hydrophobic interactions with increasing lipid concentration and the same phenomenon was also observed for the CPA value. Our results are thus in accord with these previous ones.

## 4. Conclusions

In this study, we revealed that there exists a relationship between the CPA value of EGCg and the amount of EGCg adsorbed onto a lipid-polymer membrane. In the concentration region of lipid below 1 wt% the CPA value increased with increasing lipid concentration in the membrane, and showed the highest value at 1 wt%, and then decreased beyond 1 wt%. The surface charge density and the hydrophobicity of the membrane cause the peak. The CPA value increased with increasing concentration of the EGCg solution regardless of the lipid concentration. The amounts of EGCg adsorbed onto the membrane were 0.07–7.4 μg/cm^2^ for 0.01–2.0 mM solutions. The lipid concentration dependence of the CPA value was similar to that of the amount adsorbed. The CPA value increased with increasing amount of adsorbed EGCg; however, the CPA value gradually became saturated when the amount of adsorbed EGCg increased further. As the amount of adsorbed EGCg increases the EGCg forms multiple layers, therefore, suppression of dissociation and the screening effect by paired ions occur, causing the CPA value to become saturated. These results indicate that the sensitivity of sensor can be controlled, and a taste sensor with improved functions is expected to be realized.
